# Contextual factors related to vector-control interventions for malaria: a scoping review and evidence and gap map protocol

**DOI:** 10.12688/f1000research.144661.1

**Published:** 2024-03-27

**Authors:** Timothy Hugh Barker, Grace McKenzie McBride, Mafalda Dias, Raju Kanukula, Sabira Hasanoff, Danielle Pollock, Carrie Price, Alinune Nathanael Kabaghe, Ellie A. Akl, Jan Kolaczinki, Zachary Munn

**Affiliations:** 1 Health Evidence Synthesis, Recommendations, and Impact (HESRI), The University of Adelaide, Adelaide, South Australia, Australia; 2 Albert S. Cook Library, Towson University, Towson, Maryland, USA; 3 Training and Research Unit of Excellence, Blantyre, Malawi; 4 Department of Internal Medicine, American University of Beirut, Beirut, Beirut Governorate, Lebanon; 5 Department of Health Research Methods, Evidence, and Impact (HEI), McMaster University, Hamilton, Ontario, Canada; 6 World Health Organization, Geneva, Switzerland

**Keywords:** Malaria, vector-control, acceptability, feasibility, valuation of outcomes, contextual factors

## Abstract

**Objective:**

This scoping review will identify existing literature regarding contextual factors relevant to vector-control interventions to prevent malaria. We will use the findings of the scoping review to produce an interactive evidence and gap map. The map will assist in the priority setting, development, and conduct of targeted systematic reviews. These systematic reviews seek to assist the Vector Control and Insecticide Resistance Unit of the World Health Organization’s Global Malaria Programme by informing recommendation development by their Guidelines Development Group.

**Introduction:**

Malaria contributes substantially to the global burden of disease, with an estimated 247 million cases and 619,000 deaths in 2021. Vector-control is key in reducing malaria transmission. Vector-control interventions directly target the mosquito, reducing the potential for parasite infections. These interventions commonly include insecticides used in indoor residual spraying or insecticide-treated nets and larval source management. Several new vector-control interventions are under evaluation to complement these. In addition to estimating the effects of interventions on health outcomes, it is critical to understand how populations at risk of malaria consider them in terms of their feasibility, acceptability, and values.

**Inclusion Criteria:**

Eligible studies will have assessed the contextual factors of feasibility or acceptability of the interventions of interest, or the valuation of the outcomes of interests. These assessments will be from the perspective of people who receive (residents) or deliver (workers or technicians) the vector-control intervention for the purpose of preventing malaria.

**Methods:**

We will conduct this scoping review in accordance with the JBI methodology for scoping reviews and report in line with the Preferred Reporting Items for Systematic Reviews and Meta-analyses extension for Scoping Reviews (PRISMA-ScR). We will construct the evidence and gap map following guidance from the Campbell Collaboration.

## Introduction

Malaria is a life-threatening infectious disease spread by *Anopheles* mosquitoes infected with the *Plasmodium* parasite. ^1^ In 2021 there were an estimated 247 million cases of malaria and an estimated 619,000 deaths in 84 endemic countries. ^2^ Currently, malaria case incidence sits at approximately 59 cases per 1000 people in populations at risk, with an even greater prevalence reported among pregnant women. ^2^ However, between 2000 and 2021 there was sustained and measurable progress made to combat this disease, with the number of malaria endemic countries decreasing from 108 to 84. ^2^ One of the major contributors to this decrease was the development of prevention strategies. Of these prevention strategies, ‘vector-control’ plays a critical role in the fight against the global burden of malaria. Vector-control includes interventions that are specifically designed to either target (and kill) the *Anopheles* mosquitoes, or to reduce the likelihood that populations at risk are exposed to these infected vectors. ^3^ Currently, the World Health Organization (WHO) provides explicit, evidence-based recommendations for several vector-control interventions, including insecticide-treated nets (ITNs), indoor-residual spraying (IRS), larviciding, and house modifications/screening. However, the effectiveness of these interventions varies. ^2^^–^^4^

The WHO’s Global Malaria Programme (GMP) is responsible for coordinating global efforts to control, prevent and eliminate malaria. A key component of this service involves the development of guidelines using the best-available evidence of the effects of these specific vector-control interventions on health outcomes. As stipulated in the WHO handbook for guideline development, ^5^ the WHO guideline development process requires Guideline Development Groups (GDGs) to consider two sets of factors that determine the direction and strength of a recommendation. ^5^ The first set of factors relates to the ‘health effects’ and include the evidence on the benefits (epidemiological impact) and harms of interventions, and the certainty of that evidence. ^6^ These are determined through the systematic review for appropriately selected, patient-important outcomes. ^6^ The second set of factors are ‘contextual’ and include: resource implications (is the intervention resource intensive?), equity (will the intervention reduce or increase health inequities?), feasibility of the intervention (is the intervention feasible to implement?), and acceptability of the intervention (is the intervention acceptable to key stakeholders?), as well as the valuation of the outcomes (are the health outcomes considered important by key stakeholders?). ^5^^–^^7^ These factors should all be judiciously considered by a GDG in the development of each recommendation on an intervention that appears in a WHO guideline.

As the GDG must make judgments on each of these factors, they should be well-informed regarding each of them. Synthesized evidence from relevant and rigorous systematic reviews can be a valuable source of information. ^7^ However, for some factors, other evidence sources are often considered. Evidence related to ‘equity’ is often reported in studies of the benefits and harms of interventions in terms of particular groups who may (or may not) benefit from the intervention or who may be overlooked in the rollout of interventions. ^8^ This type of data can sometimes be sourced from quantitative studies if they report PROGRESS-Plus criteria. ^8^^,^^9^ The remaining factors, ‘resource implications’, ‘feasibility’, ‘acceptability’, and ‘valuation of outcomes’, may also be informed by a rigorous systematic review of the appropriate and relevant primary studies. ^6^

There has been a concerted effort by the WHO GMP to update its guidelines on vector-control interventions using systematically collected evidence. However, there has been no previous scoping review on the contextual factors associated with vector-control initiatives. In this protocol, we outline the protocol of a scoping review and an evidence and gap map to provide the WHO with an overview of the quantity and type of studies available addressing the contextual factors of ‘feasibility’ and ‘acceptability’ of specific vector-control interventions, and of ‘valuation of the outcomes of interest’. Given that considerations associated with the factor of ‘resource implications’ (e.g., costs, resources, cost-effectiveness etc.) are specific to the site/study/time-point under investigation, this factor can be assessed in many, and often inconsistent, ways. Therefore, the production of a map of this evidence was determined to not be useful, and as such it will not be considered as part of this scoping review.

This scoping review, and the production of the proposed evidence and gap map will enable the WHO to consider whether to commission future, targeted systematic reviews or primary studies in these areas.

## Review question

What evidence exists regarding the ‘feasibility,’ and ‘acceptability of specific vector-control interventions for the prevention of malaria’ and the ‘valuation of the outcomes of interest’?

## Methods

Evidence and gap maps are described as “a systematic presentation of all relevant evidence of a specified kind for a particular sector, sub-sector, or geography”. ^10^ The proposed scoping review will be conducted in accordance with the JBI methodology for scoping reviews. ^11^^,^^12^ It will be reported in line with the Preferred Reporting Items for Systematic Reviews and Meta-Analyses (PRISMA) extension for Scoping Reviews (PRISMA-ScR). ^13^ The search will be peer reviewed by another information specialist using the PRESS Guidelines criteria and appropriate revisions included. ^14^ The evidence and gap map will be constructed following the guidance from the Campbell Collaboration. ^15^ This review is appropriate to be conducted as a scoping review, as it aims to identify the type of evidence available in the field of malaria vector-control and to examine how research is conducted on that topic. ^16^

## Eligibility criteria

### Participants

Studies will be eligible for inclusion where they have investigated the concepts of this scoping review (below) in adults and children who are residents, or part of nomadic communities, of a region with ongoing malaria transmission. Studies will also be eligible for inclusion where they have investigated the concepts of this scoping review in deliverers (workers or technicians) of a vector-control intervention. Studies regarding experimental hut trials for vector-control interventions that use human sleepers will also be included, where the study relates to the participants’ acceptability of the intervention.

### Contextual factors

Studies will be eligible for inclusion in this scoping review where they addressed the contextual factors of feasibility, acceptability, and valuation of outcomes of participants (defined in the introduction) in response to a vector-control intervention as listed below.

This review will explicitly not consider studies that investigate the contextual factors of ‘resource implications’ or ‘equity’ of a vector-control intervention. ‘Resource implications’ can be site/study/time-point specific and assessed in many, and often inconsistent, ways. This information may also be contained in the studies evaluating the benefits and harms of an intervention. However, studies that mention equity implications relating to ‘acceptability,’ ‘feasibility,’ or ‘valuation of the outcomes of interest’ will be identified with our search. Studies with information on factors related to vector-control interventions, such as factors adjunct to the intervention that may influence the implementation strategy, will be included. These factors may include the durability of the vector-control intervention, and education programs run alongside deployment of the intervention, which may impact the acceptability or feasibility of the intervention.

### Vector-control interventions

Studies will be eligible for this scoping review where they have investigated the above contextual factors in response to the implementation of one of the following vector-control interventions:

• Insecticide-treated nets
• Indoor-residual spraying
• Outdoor-residual spraying
• House screening and other housing modifications for malaria prevention
• Eave tubes
• Spatial repellents
• Endectocides
• Mosquito traps
• Topical repellents
• Larval source management practices (i.e., larviciding, habitat modification, habitat manipulation or biological control by means of natural predators of mosquito larvae)
• Attractive targeted sugar baits
• Vector-control interventions not specified above (i.e., insecticide-treated clothing or hammocks, insecticide treated wall linings and genetically modified mosquitoes amongst others yet to be identified)

### Context

This scoping review will only consider the above concept if it was investigated as part of, or alongside the deployment of, one or more vector-control interventions for malaria prevention. Where the intervention has been used to prevent other vector-borne diseases, these studies will not be considered eligible. Finally, contextual factors related to other interventions utilized for malaria prevention or treatment (e.g., vaccination, mass drug administration and education programs etc.) will also not be considered. Only studies that have provided data regarding vector-control interventions will be eligible for this review. The presence of other background interventions will not impact on study eligibility as long as they are ubiquitous within the study population (e.g., ITN installation with ubiquitous education programs). There will be no exclusions based on publication date, language or publication status (i.e., published, in press, in progress, pre-print). For studies published in languages other than English, we will use DeepL Translator ^17^ to determine whether the study meets the inclusion criteria. Where studies are published in a language other than English and meet the inclusion criteria, DeepL translations will be reviewed by a person fluent in the language.

### Types of documents

This scoping review will only consider peer-reviewed research articles of any methodology. Due to the nature of contextual factors research, studies may utilize diverse methods in their data collection and no restriction will be placed on the types of studies considered eligible.

### Search strategy

The search strategy aims to locate both published and unpublished studies and was developed with the input of a medical librarian.

An initial limited search of PubMed via NCBI was undertaken to identify relevant articles on this topic. The terminology contained in the titles and abstracts of relevant articles, as well as related subject headings, will inform a full search strategy. The search strategy, including all identified keywords and subject headings, will be adapted for each included database or information source, by using Polyglot ^18^ and with the aid of a medical librarian. The databases to be searched include PubMed (NCBI [contains MEDLINE]), Embase (Elsevier), Cochrane Database of Systematic Reviews (CDSR; Wiley), the Cochrane Central Register of Controlled Trials (CENTRAL; Wiley), Scopus (Elsevier) and WHO Global Index Medicus. The full search strategy for PubMed (NCBI), and the results of this search are available online.

### Study selection and screening

Following the search, all identified records will be collated and uploaded into EndNote ^TM^ . Duplicates will be removed using the Deduplicator in the Systematic Review Accelerator. ^19^ Records will then be imported into Covidence where any remaining duplicates will be identified and removed. All records will then be screened on their titles and abstracts by two or more independent reviewers against the eligibility criteria for the review. A record without an abstract will be included for full-text retrieval. Piloting of this screening process will take place on 5% (or minimum five records) of the records imported into Covidence. Piloting of screening will take place between all reviewers. Results of the piloting will then be compared; more piloting will occur if agreement reached is less than 70% between all reviewers. Following piloting, the remaining records will be screened at the title and abstract level independently, so that each record will be screened by at least two people.

Potentially relevant records will be exported from Covidence, and the full text of each record will be retrieved. The records will then be imported to EppiReviewer. ^20^ The full text of each relevant record will be assessed against the eligibility criteria by two or more independent reviewers. Another pilot test will occur on 5% (or minimum five reports) of the reports included for screening at the full-text level. Additional piloting will occur if agreement reached is less than 70% between all reviewers. Following piloting, the remaining reports will be screened in detail against the inclusion criteria by two or more independent reviewers, so that each report is screened by at least two people. For reports that do not meet the eligibility criteria after being screened at the full-text level, the reasons for their exclusion will be documented and reported in the appendices of the final scoping review. Any disagreements that arise between the reviewers at any stage of the selection process will be resolved through discussion, or with additional reviewers. The results of the search and screening process will be reported in full in the final review and presented in a PRISMA 2020 flow diagram. ^21^

Reasons for exclusion at the full-text level may be due to:

• Conference abstract (no associated full text)
• Study protocol
• Expert opinion/editorial/letter
• Not malaria
• Not contextual factor of interest
• Not vector-control intervention
• Not appropriate population/setting (e.g., modelling study).

### Data extraction and coding

All studies that meet the eligibility criteria following screening at the full-text level will undergo data extraction within EppiReviewer. Data will be extracted from all eligible studies by two or more independent reviewers. Piloting and training of the reviewers on the extraction form as it exists within EppiReviewer will occur, and a data dictionary of terms will be produced and iteratively updated to guide extractors (described below). Any disagreements between reviewers during extraction will be resolved through discussion or consultation with a third reviewer who was not involved in the original extraction. If appropriate, authors of papers will be contacted to request missing or additional data where required. The data will firstly be extracted according to the demographic characteristics of the study and include the following items:

• First author name
• Year of publication
• Country/city/region in which the study took place
• Year/month the study took place.

Following this, the studies will be coded within EppiReviewer. Coding will be based on the following three categories, and structured to generate the evidence and gap map (described below):

1. Vector-control intervention
  a. Insecticide-treated net
  b. Indoor-residual spraying
  c. Outdoor-residual spraying
  d. House improvements/screening
  e. Eave tubes
  f. Spatial repellents
  g. Mosquito traps
  h. Endectocides
  i. Topical repellents
  j. Larval source management practices
  k. Attractive targeted sugar baits
  l. Vector-control intervention not specified by the WHO.
2. Contextual factors
  a. Acceptability (of the intervention(s) of interest) from the perspective of:
    i. The public
    ii. The deliverer
  b. Feasibility (of the intervention(s) of interest) from the perspective of:
    i. The public
    ii. The deliverer
  c. Valuation of the outcomes of interest (i.e., potentially affected by the interventions of interest).
  d. Factors related to the intervention.
3. Study methods
  • Experimental studies
  • Observational studies
  • Utility/health status studies
  • Close-ended questionnaires/surveys
  • Open-ended questionnaires/surveys
  • Focus groups
  • Interviews
  • Literature review
  • Systematic review
  • Scoping review.

### Data dictionary

To facilitate a common and shared language between extractors before and during data extraction and coding, a data dictionary of terms will be developed and updated iteratively as new concepts are identified, explored and agreed upon by the review team ( Table 1). This practice will ensure that concepts of similar meaning and definitions are extracted and coded consistency between multiple extractors. Due to the iterative nature of developing a data dictionary, new terms are likely to be added to this repository using the inductive coding functions available in EppiReviewer. As such, the below should only serve as an example of the current terms considered as part of the data dictionary.

**Table 1.** Data dictionary of terms. This is an example of the development of a data dictionary that will take place during the extraction and coding of the studies considered eligible for this review.

Term	Concept	Definition
Insecticide-treated net	Vector-control intervention	Mosquito net that repels, disables or kills mosquitoes that come into contact with the insecticide on the netting material. Insecticide-treated nets (ITNs) include those that require treatment and retreatment (often referred to as conventional nets) and those that are “long-lasting”. ^22^
Indoor-residual spraying	Vector-control intervention	Spraying the interior walls and ceilings of dwellings with a residual insecticide to kill or repel endophilic mosquito vectors of malaria. ^22^
Outdoor-residual spraying	Vector-control intervention	Spraying the exterior walls and ceilings of dwellings with a residual insecticide to kill or repel endophilic mosquito vectors of malaria. ^23^
House screening/modification	Vector-control intervention	Covering of housing (either completely, or eaves of the household) with a physical barrier to reduce mosquito entry to the home. ^24^ This will also include any modifications made to the home in an effort to control vectors.
Eave tubes	Vector-control intervention	A physical barrier that does not prevent air-flow, placed over eaves of households and buildings, in which insecticide-treated nettings are inserted. ^25^
Spatial repellents	Vector-control intervention	Any substance that causes avoidance in mosquitoes, by deterring them from entering an area or room and/or disrupt their human biting or feeding habits. ^22^
Mosquito traps	Vector-control intervention	Devices designed for capturing mosquitoes with or without attractant components (e.g., light, CO _2,_ living baits, sugar, suction). ^22^
Endectocides	Vector-control intervention	Drugs used in livestock and agricultural industries to control endoparasite and ectoparasite vectors. ^26^
Topical repellents	Vector-control intervention	Any substance that causes avoidance in mosquitoes, by deterring them from settling on the skin of the host. ^22^
Larval source management	Vector-control intervention	Management of aquatic habitats (water bodies) that are potential habitats for mosquito larvae, in order to prevent completion of development of the immature stages of the larvae. ^22^ The four types of larval source management are: habitat modification, which is a permanent alteration of the environment, e.g. land reclamation; habitat manipulation, which is a recurrent activity, e.g. flushing of streams; larviciding, which is regular application of biological or chemical larvicides to water bodies; and biological control, which consists of the introduction of natural predators of mosquito larvae into water bodies. ^22^
Attractive targeted sugar baits	Vector-control intervention	Attractive Targeted Sugar Baits (ATSB) have three components, an attractant, sugar and an active ingredient that kills mosquitoes. A protective membrane covers and protects the bait from rain, dust and serves as a barrier to pollinators but allows mosquitoes to feed through it. ^27^^,^^28^
VCI not specified by the WHO	Vector-control intervention	Where a study of a VCI has been identified during the search that examines the impact of a VCI not prespecified by the WHO in regard to the contextual factors as described below, the VCI for this study will be coded as such. Any VCIs that involve the use of genetically modified mosquitos will be defined as per the ‘Guidance Framework for Testing Genetically Modified Mosquitoes’ Second Edition’. ^29^
Acceptability	Contextual factor	Is the option acceptable to key stakeholders? ^7^ Acceptability might reflect who benefits (or is harmed) and who pays (or saves); and when the benefits, adverse effects, and costs occur (and the discount rates of key stakeholders; e.g. politicians may have a high discount rate for anything that occurs beyond the next election). Unacceptability may be due to; • Not accepting the distribution of the benefits, harms, and costs • Not accepting costs or undesirable effects in the short term for desirable effects (benefits) in the future • Attaching more value (relative importance) to the undesirable consequences than to the desirable consequences or costs of an option (because of how they might be affected personally or because of their perceptions of the relative importance of consequences for others) • Morally disapproving (i.e. in relationship to ethical principles such as autonomy, nonmaleficence, beneficence or justice)
Feasibility	Contextual factor	Is the option feasible to implement? ^7^ Can the option be accomplished or brought about? The less feasible (capable of being accomplished or brought about) an option is, the less likely it is that it should be recommended (i.e. the more barriers there are that would be difficult to overcome). Feasibility can overlap with valuation of outcomes, resource considerations, existing infrastructure, equity, cultural norms, legal frameworks, and many other considerations. ^5^^,^^7^
Valuation of the outcomes of interest	Contextual factor	This describes the relative importance assigned to health outcomes by those affected by them, how such importance varies within and across populations, and whether this importance or variability is surrounded by uncertainty. ^5^ When used generically as in “values and preferences” we refer to the collection of goals, expectations, predispositions, and beliefs that individuals have for certain decisions and their potential outcomes. ^30^
Factors related to the intervention	Contextual factor	This includes studies that have reported on factors that exist adjunct to the intervention and may have some influence on the implementation strategy of the intervention. These include considerations such as (but not limited to) education programs that have been deployed that demonstrated change in how the intervention is accepted among the community. It can also include concerns related to the physical integrity of the intervention, such as durability or longevity considerations that may influence how the intervention is implemented in the real-world setting.
Experimental studies	Study method	Include randomized controlled trials and their derivatives (cluster-randomized controlled trials, cross-over trials etc.), pseudo-randomized controlled trials and quasi-experimental studies. ^31^
Observational studies	Study method	Include prospective and retrospective cohort studies, case-control studies, analytical cross-sectional studies and case series/case reports. ^32^
Utility/health status value studies	Study method	Include the following types of studies: standard gamble, time trade off, discrete choice, visual analogue scale, multi-attribute instruments, utility or health status values transformed from quality of life measurements. ^33^
Close-ended questionnaires/surveys	Study method	Include surveys/questionnaires in which questions are presented that have an explicit/discrete/guided answer that is to be selected by the particpants. ^34^
Open-ended questionnaire/surveys	Study method	Include surveys/questionnaires in which questions are presented with no explicit/discrete/guided answer associated, and allow the participant to reflect personally on the question being asked. ^34^
Focus groups	Study method	Include group interviews that capitalize on communication between research participants and the researcher to generate data. ^35^
Interviews	Study method	Include semi-structured or unstructured communication between a single research participant and the researcher to generate data. ^36^
Literature review	Study method	Traditional literature reviews are useful for describing an issue and its underlying concepts and theories. However, they rely heavily on the author's knowledge and experience and provide a limited presentation of a topic. Such reviews are often based on references chosen selectively from the evidence available, resulting in a review inherently at risk for bias or systematic error. ^37^
Systematic review	Study method	Systematic reviews seek to collate evidence that fits pre-specified eligibility criteria to answer a specific research question. They aim to minimize bias by using explicit, systematic methods documented in advance with a protocol. ^38^
Scoping review	Study method	Scoping reviews may be conducted where the purpose of the review is to identify knowledge gaps, scope a body of literature, clarify concepts or to investigate research conduct. While useful in their own right, scoping reviews may also be helpful precursors to systematic reviews and can be used to confirm the relevance of inclusion criteria and potential questions. ^39^

### Data analysis and presentation

All extracted data, regardless of presentation in the evidence and gap map as described below, will be narratively synthesized and presented in tables, with further visual formats to be considered at the time of analysis, to provide frequency counts and descriptive statistics of the following items.

• When and where the studies were conducted
• The types and formats of the bodies of evidence identified
• The vector-control interventions investigated
• The contextual factors investigated.

### Evidence mapping

**Figure 1.**
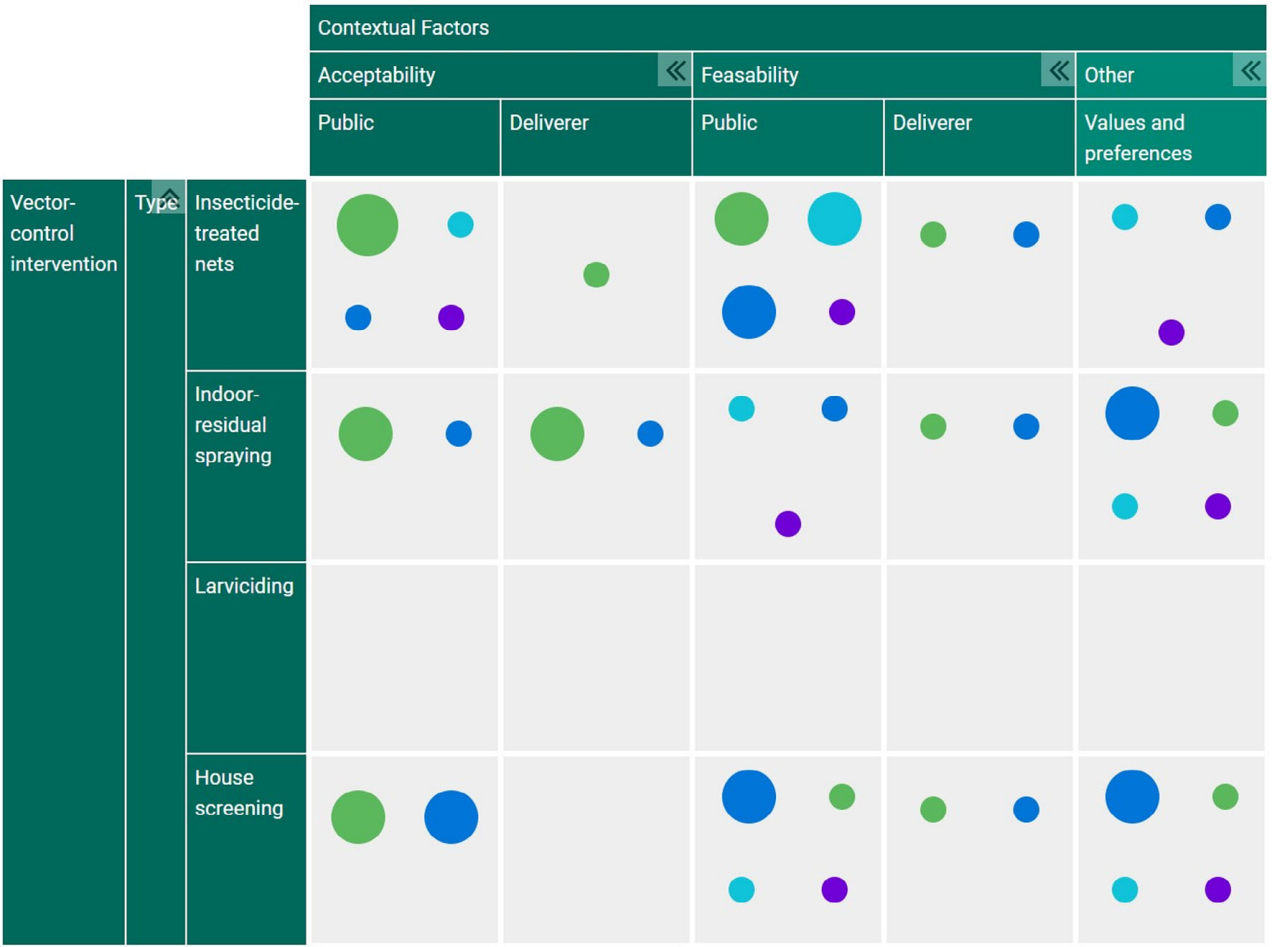
Example of the evidence and gap map that will be produced following this scoping review. The data has been organized based on the contextual factors of interest (columns) against the vector-control interventions of interest (rows). Each colored circle represents the study methods used, with the size of the circle indicating how many studies exist for each category. Green = quantitative studies, Aqua = Qualitative studies, Blue = mixed-methods studies, Purple = evidence reviews. This figure was produced by the authors of this study as an example only.

The results of the data extraction and coding will be presented in an evidence and gap map that will be developed using the EppiMapper software v4.0. ^40^ The evidence and gap map will be organized according to the data coding hierarchy as presented above. An example of what the final evidence and gap map will appear as is presented in Figure 1.

### Ethics and dissemination

This scoping review is a review of primary and secondary studies and as such, does not require ethics approval. In addition, there will be no collection or analysis of individual participant data. All study files (data extraction forms and EppiReviewer.json files) will be made publicly available via a project space using open science platforms. A full review report will be submitted to the Vector Control & Insecticide Resistance Unit, Global Malaria Program, and the Vector Control Guidelines Development Group, WHO, to inform potential targeted systematic reviews to be undertaken on these factors in the future. A version of this report will be submitted for publication in an open access peer-reviewed journal.

### Stakeholder engagement

This scoping review is being conducted for the purposes of informing future WHO systematic reviews and guideline development. Guideline development panel members, which include various diverse stakeholders including the public, patients, end users, experts and decision-makers may guide the interpretation and implementation of the results of this scoping review.

### Study status

This study has been completed.

## Contributorship statement

Conceptualization: THB, ZM, EAA, JK and colleagues from the Global Malaria Program, WHO

Writing – Original draft preparation: THB, GMM, MD, RK, SH, DP, ANK, ZM

Data curation: CP led the development of the search strategy.

Writing – Review and editing: THB, GMM, MD, RK, SH, DP, CP, ANK, EAA, JK, ZM

## Data Availability

No data are associated with this article.

## Software statement

Covidence, EppiMapper, and the Systematic Review Accelerator software are freely available. Endnote is a paid software platform. A free equivalent referencing software is Mendeley. EppiReviewer is a paid platform. Rayyan is available as a free alternative.
