# Supplementary material for: Contextual factors related to vector-control interventions for malaria: a scoping review and evidence and gap map protocol
Source: F1000Res. 2024 Mar 27;13:226. [Version 1] doi: 10.12688/f1000research.144661.1 (PMC11214036; doi:10.12688/f1000research.144661.1)
Supplement: Peer review history

# Reviewer report: Reviewer response for version 1

**DOI:** 10.5256/f1000research.158491.r289708

**Published:** 2024-06-28

**Authors:** Justin Pulford

**Affiliations:** 1 Department of International Public Health, Liverpool School of Tropical Medicine, Liverpool, UK

This paper outlines the protocol for a planned scoping review and evidence and gap map intended to provide the WHO with an overview of the quantity and type of studies available addressing the contextual factors of ‘feasibility’ and ‘acceptability’ of specific vector-control interventions, and of ‘valuation of the outcomes of interest’. The study objectives are clearly described, the study design is appropriate and sufficiently detailed.  Overall, this is a very well written protocol.  I only have a few questions and or suggested edits to make, all of which I would consider minor:

 Introduction

 The authors define five sets of ‘contextual’ factors: resource implications, equity, feasibility, acceptability and valuation.  The authors then state that their planned scoping review will only focus on the last three of these factors, excluding resource implications and equity.  Clear and reasonable justification is given for excluding ‘resource implications’, but the justification for excluding ‘equity’ remains somewhat unclear.  Are the authors contending that equity cannot be appropriately assessed via systematic review or that it is unnecessary to do so because equity considerations can be derived from other sources? If the latter, the argument put forth appears somewhat weak (as could be true of all factors) and is also perhaps better placed in the paragraph below alongside the justification for excluding ‘resource implications’.

 Methods

 Data extraction:

 There might also be value in recording the institutional affiliation of the first authors. This would provide some insight into which countries are leading the research effort.

 Might there also be value in coding whether the ‘valuation of the outcomes of interest’ comes from ‘the public’ or ‘the deliverer’?  This would be consistent with the coding approach for the other two factors and seems like an important element to capture.

 What is meant by ‘d. factors related to the intervention’?  This is not clear without an accompanying explanation.

 Why have codes for ‘systematic review’ and ‘scoping review’ been included?  Wouldn’t papers of this type have been excluded from review?

 Will multiple responses to ‘3.study methods’ codes be allowed?  E.g. a study questionnaire could include both open and closed questions or a study could include both surveys and interviews

Is the study design appropriate for the research question?

Yes

Is the rationale for, and objectives of, the study clearly described?

Yes

Are sufficient details of the methods provided to allow replication by others?

Yes

Are the datasets clearly presented in a useable and accessible format?

Not applicable

Reviewer Expertise:

Malaria control/vector control

I confirm that I have read this submission and believe that I have an appropriate level of expertise to confirm that it is of an acceptable scientific standard.

# Reviewer report: Reviewer response for version 1

**DOI:** 10.5256/f1000research.158491.r278283

**Published:** 2024-05-31

**Authors:** Salum Azizi

**Affiliations:** 1 Department of Medical Parasitology and Entomology, Kilimanjaro Christian Medical University College, Moshi, Tanzania

This protocol is well prepared to provide information on quantity and type of studies available that addresses contextual factors of ‘feasibility’, ‘acceptability’ and ‘valuation of the outcomes of interest’  to facilitate evidence-based recommendation on vector control interventions by the WHO.

 However, the author should consider addressing the following minor issues:

1. Rephrase the statement “These interventions commonly include insecticides used in indoor residual spraying or insecticide-treated nets and larval source management” as it sounds as if insecticides on iself is an intervention. The interventions here are indoor residual spraying, insecticide treated nets and larvae source management which uses insecticide or might not eg larvae source management might includes other strategies such as habitat modification or manipulation without necessarily involving insecticides.
2. By excluding resource implications which is very much contributing to intervention feasibility, a clear definition and limitation for term “feasibility” is needed. The current definition in the introduction does not clearly provide information what it entails.
3. Add ethical violations among the reasons for exclusion at the full-text level articles
4. Figure 1. Correct the term “feasability” to “feasibility”

Is the study design appropriate for the research question?

Yes

Is the rationale for, and objectives of, the study clearly described?

Yes

Are sufficient details of the methods provided to allow replication by others?

Yes

Are the datasets clearly presented in a useable and accessible format?

Not applicable

Reviewer Expertise:

Evaluation of mosquito control interventions

I confirm that I have read this submission and believe that I have an appropriate level of expertise to confirm that it is of an acceptable scientific standard.
